# Current Concepts on 6-sulfo LacNAc Expressing Monocytes (slanMo)

**DOI:** 10.3389/fimmu.2019.00948

**Published:** 2019-05-22

**Authors:** Fareed Ahmad, Thomas Döbel, Marc Schmitz, Knut Schäkel

**Affiliations:** ^1^Department of Dermatology, Heidelberg University Hospital, Heidelberg, Germany; ^2^Dermatology Branch, National Institute of Arthritis and Musculoskeletal and Skin Diseases, Bethesda, MD, United States; ^3^Institute of Immunology, Faculty of Medicine Carl Gustav Carus, Technische Universtät Dresden, Dresden, Germany; ^4^Partner Site Dresden, National Center for Tumor Diseases (NCT), Dresden, Germany

**Keywords:** slan^+^ monocytes, slanMo, non-classical monocytes, inflammation, autoimmunity, cancer, infection, psoriasis

## Abstract

The human mononuclear phagocytes system consists of dendritic cells (DCs), monocytes, and macrophages having different functions in bridging innate and adaptive immunity. Among the heterogeneous population of monocytes the cell surface marker slan (6-sulfo LacNAc) identifies a specific subset of human CD14^−^ CD16^+^ non-classical monocytes, called slan^+^ monocytes (slanMo). In this review we discuss the identity and functions of slanMo, their contributions to immune surveillance by pro-inflammatory cytokine production, and cross talk with T cells and NK cells. We also consider the role of slanMo in the regulation of chronic inflammatory diseases and cancer. Finally, we highlight unresolved questions that should be the focus of future research.

## CD16^+^ Monocytes

Monocytes are important regulatory cells in innate and adaptive immunity (1, 2). Studies on blood leukocytes showed that monocytes are a heterogeneous cell population that can be roughly separated into three populations: classical monocytes CD14^+^CD16^−^, intermediate monocytes CD14^+^CD16^+^, and non-classical monocytes CD14^−^CD16^+^ (1, 3–5). The murine counter part of non-classical monocytes was identified as Ly6C^low^CCR2^−^CX3CR1^hi^ cells (4, 6, 7). So far the most distinctive and best-studied function of mouse non-classical monocytes is their migration independent of the direction of blood flow along the luminal side of the vascular endothelium (8–10). There, they function in immune surveillance of the vasculature and exert both anti-inflammatory and pro-inflammatory functions. Therefore, they are also called patrolling monocytes (8). Patrolling behavior is a common feature of both murine and human non-classical monocytes (8, 9, 11, 12). However, murine non-classical monocytes are currently considered to be cells of vascular homeostasis, while the majority of studies describe an overall pro-inflammatory function of human non-classical monocytes (9, 11, 13, 14). The pro-inflammatory function of human non-classical monocytes is mainly attributed to the production of TNF-α and IL-12 (6, 11, 15–17). Concerning the origin of non-classical monocytes, there is now evidence from studies in mice and humans that classical monocytes give rise to non-classical monocytes (18, 19). The transcription factor Nur77 (NR4A1) is upregulated in human and murine non-classical monocytes (17, 19, 20). Mice, having a deletion in the NR4A1 super enhancer, lack non-classical monocytes and serve as a model to study their function *in vivo* (10).

In the absence of specific markers, studies on human CD16^+^ monocytes are largely descriptive, and rely on CD14/CD16-gating strategies with no clear-cut definition. Numbers of intermediate monocytes and non-classical monocytes in blood were found altered under various conditions (15, 21–25). These studies are confined to blood leukocytes, as there is no stable expression of CD14 and CD16 on non-classical monocytes entering into tissues or differentiating into macrophages and DCs.

Within human CD14^−^CD16^+^ non-classical monocytes, our group defined a 6-sulfo LacNAc (slan) expressing cell population (slanMo) in peripheral blood (16, 26, 27). Subsequently, slan expressing cells have been identified in tissues (16, 28–31). Therefore, the stably expressed slan antigen provides a unique opportunity to study these cells in different organs.

## Identity of slanMo Expressing Cells

slanMo research began in 1998 when a CD16^+^ cell population accounting for 50% of non-classical monocytes was defined by the mAb M-DC8 (32, 33). The mAb M-DC8 (IgM) was generated by immunizing mice with peripheral blood mononuclear cells (PBMCs), depleted of CD14^+^ monocytes, T cells and B cells (33). DD1 and DD2 (IgM, generated by immunization with slanMo) are additional slan-specific mAbs that allowed for the detection of slan^+^ cells in paraffin-embedded tissue sections (30, 31, 34). slanMo specifically express the eponymous “slan” antigen (6-sulfo LacNAc), an O-linked glycosylated variant of P-selectin glycoprotein ligand-1 (PSGL-1) (25, 30). At the molecular level, the slan-antigen is a non-sialylated and non-fucosylated 6-sulfated N-acetyllactosamine (LacNAc) (26). This is in contrast to the cutaneous lymphocyte-associated antigen (CLA, also known as sialyl 6-sulfo Lewis X), which is a sialylated and fucosylated variant of 6-sulfo LacNAc. While CLA binds to E-selectin and thereby facilitates skin homing of T cells, slan was shown to be devoid of binding to E- and -L-selectin (35). The exact function and the binding partners of slan are unknown. However, sulfated terminal glycotopes as found in the slan-antigen were shown to serve as ligands for lectins other than E- and–L-selectin, including members of the galectins and siglec families (36–41).

Transcriptomic studies on blood leukocytes clearly identified slan^+^ cells as a subset of monocytes and accordingly they were called slanMo (4, 11, 42, 43). While being of monocyte origin, slanMo may either rapidly acquire dendritic cell functions (4, 42, 44) or differentiate into macrophages (29, 45). Their initial recognition as dendritic cells (DCs) (33) was based on their DC-like phenotype with very low or undetectable levels of the classical monocytes markers CCR2, CD14, CD62L, CD11b, and CD36 as well as their function as professional antigen presenting cells as revealed by T cell stimulatory experiments (16, 30). Similarly, in skin tissue of psoriasis patients, slan^+^ cells showed a DC-like phenotype (CD14^−^, CD163^−^) and function (IL-23p19^+^) (30).

slanMo purified from human tonsil tissue resembled DCs by morphology and function (28). They co-localized with T cells in tonsils and induced their proliferation several times more efficient than macrophages and similar to bona fide DCs (DC1, DC2, and pDC). In addition, peripheral blood slanMo cultured in tonsil-derived condition medium acquired the phenotype of slanMo in tonsils (28). slan^+^ cells in lymph nodes of patients with diffuse large B-cell lymphoma, exhibited a phenotype of either immature DCs (CD163^low^/CD14^low^/CD64^low^/CD16^low^) or macrophages (CD163^hi^/CD14^hi^/CD64^hi^/CD16^hi^) (29). Furthermore, *in vitro* studies revealed that GM-CSF and IL-4-treated slanMo can differentiate into cells with a DC-like phenotype, while IL-34-treated slanMo revealed a macrophage-like phenotype (28). Thus, slanMo may be considered as a type of circulating and tissue myeloid cell population with remarkable plasticity (28, 29, 46).

Recently, Hamers et al. defined heterogeneity within human monocytes (Table 1) using mass cytometry combined with single cell sequencing data (47). slanMo, but not slan-negative non-classical monocytes, were shown to express CXCR6, which facilitated chemotactic migration toward CXCL16 (47, 48). Interestingly, CXCL16 was previously shown to be upregulated in psoriasis, lupus nephritis as well as in cardiovascular disease (47, 49–52). In line with this study describing slanMo as having phenotype and functions distinct from other non-classical monocytes, Hofer et al. reported on a selective depletion of slan-negative CD16^+^ cells in patients with sarcoidosis (53). Furthermore, they demonstrated a 5-fold depletion of slan-positive monocytes in patients with hereditary diffuse leukoencephalopathy with axonal spheroids (HDLS), a disease caused by macrophage colony-stimulating factor (M-CSF) receptor mutations.

**Table 1:**
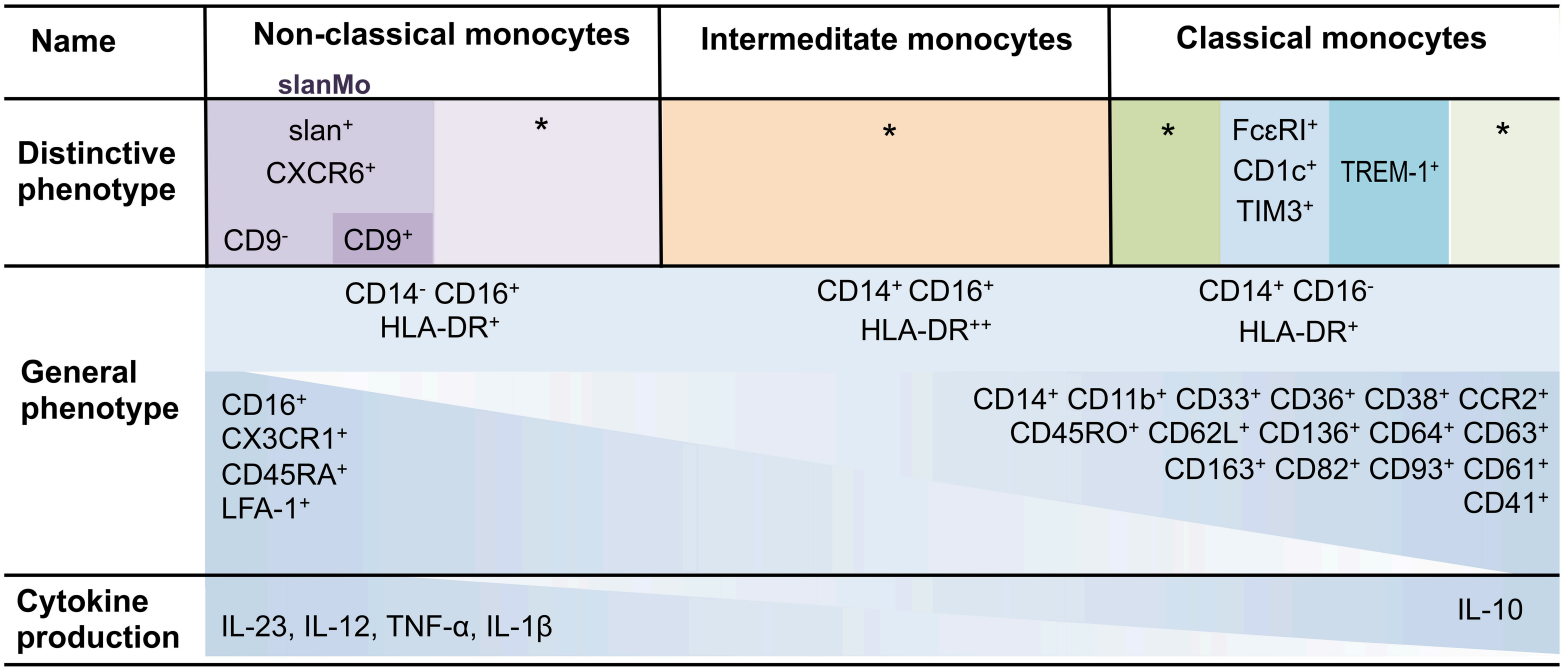
Human monocyte heterogeneity.

^*^Defined by a rather complex set of differentially expressed molecules (47). The table summarizes phenotypic and functional aspects of human monocyte heterogeneity. According to previous work and the recent study, 4 classical, 1 intermediate, and 3 non-classical monocyte populations can be defined (represented by different color code) (47). The 3 non-classical monocyte populations are identified by the differential expression of slan and CD9. General differences in phenotype and cytokine production are depicted as well. Intensity of color represent the expression level of surface marker and cytokines production from non-classical monocytes to classical monocytes and vice-versa.

## Function of slanMo

**Figure 1:**
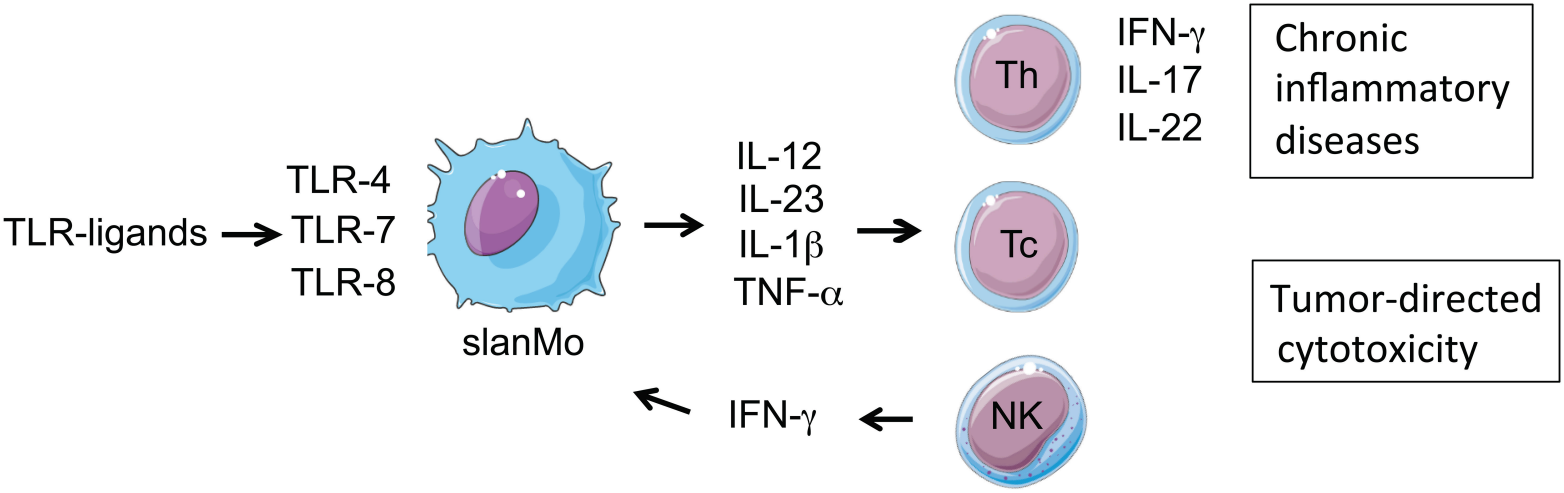
Immune regulatory function of slanMo: slanMo are activated via TLR stimulation to produce pro-inflammatory cytokines, thereby programming and enhancing Th1 and Th17 T cell responses, which play a major role in chronic inflammatory diseases. Activated slanMo also promote cytotoxic CD8 and NK cell-mediated anti-tumor responses. In a positive forward feedback loop slanMo producing IL-12 stimulate NK cells for early production of IFN-γ, which amplifies IL-12 production by slanMo.

**Figure 2:**
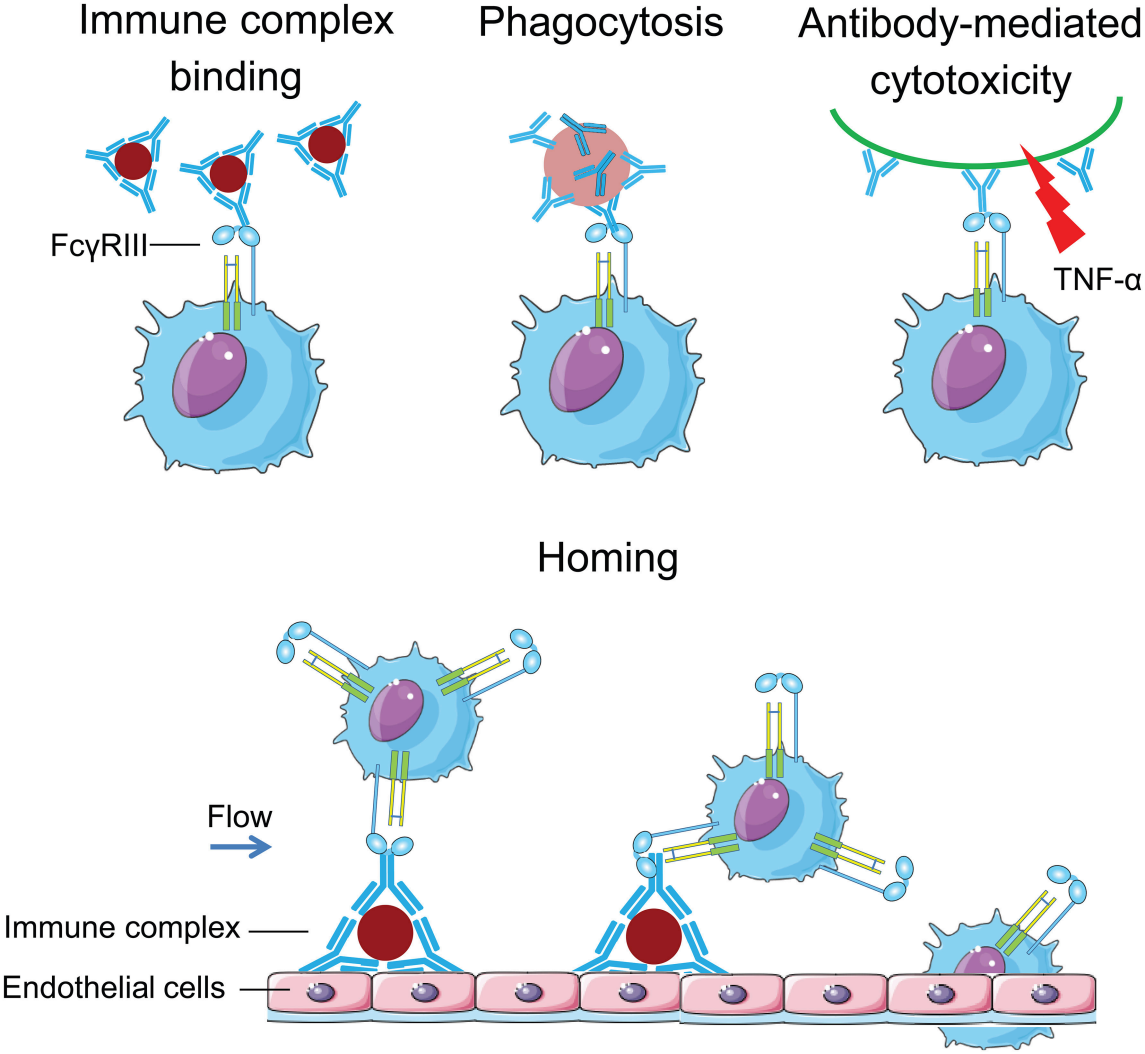
CD16 equips slanMo with a strong capacity to handle complexed IgG: slanMo can bind to IgG immune complexes through CD16. They can also phagocytose and mediate antibody-mediated cellular cytotoxicity (ADCC) after binding of CD16 to antibody (IgG)- coated cells. This is in difference to monocytes expressing CD32 for engaging immune complexes. Moreover, slanMo in the blood flow can home to immobilized vascular immune complexes via CD16.

The selective slan-marker opened the possibility for functional studies (Figures 1, 2) after mAb-directed purification of slanMo. In blood, slanMo circulate as cells with low-level expression of HLA-DR and co-stimulatory molecules (26, 30, 47). They express a broad range of toll-like receptors (TLRs) but lack TLR3 and TLR9 (46). Stimulation of freshly isolated or immature slanMo with lipopolysaccharide (LPS) or CD40 ligand resulted in high-level TNF-α production (16, 26, 54). TNF-α-producing slanMo were identified in psoriasis, lupus skin lesions, glomerular capillaries of lupus nephritis, and tumor draining lymph nodes (30, 31, 43, 46). Stimulating freshly isolated slanMo did not induce IL-12 or IL-23 production (11, 26, 42) however, slanMo revealed an outstanding capacity to produce IL-12 and IL-23 compared to blood monocytes and DCs, when stimulated after a brief culture period of 6 h (16, 31, 44). This functional maturation occurred when slanMo were left unseparated as in whole PBMC cultures and also after their purification by slan-directed magnetic cell sorting. The phenotypic maturation was reflected by upregulation of CD83, CD80, and HLA-DR, while CD16 was shed from the surface by activation of a disintegrin and metalloproteinase domain-containing protein 17 (ADAM17) (16). During this maturation process, expression of the slan antigen remained stable. Interestingly, maturation of slanMo could be completely prevented when erythrocytes were added to *in vitro* cultures of already purified slanMo. Therefore, in peripheral blood, maturation of slanMo may be tightly controlled by circulating erythrocytes (16). mAb-directed blocking experiment revealed that the inhibitory effect of erythrocytes depended on the expression of CD47 on erythrocytes and its binding to signal-regulatory protein α (SIRPα) on slanMo (16). The *in vitro* findings of slanMo producing TNF-α and IL-23 are mirrored by studies on psoriasis skin lesions where 85% of dermal slanMo were found to express IL-23p19 and 50% of the cells expressed TNF-α (31). Conditioning slanMo with IFN-γ for 6 h before stimulation with LPS or R848 increased (10-fold) their IL-12 secretion acknowledging the relevance of a positive feedback loop with IFN-γ producers such as Th1 cells and NK cells (55). slanMo revealed a strong response to TLR7 and TLR8 ligands with high IL-12 and IL-23 production (31, 54). Interestingly, IL-23 production required autocrine signaling by TNF-α and IL-1β (56, 57). The responsiveness to TLR7 and TLR8 stimulation is relevant for the activation of slanMo in autoimmune diseases and psoriasis where single stranded RNA motives are either contained within autoimmune complexes or being complexed by the antimicrobial peptide LL37 as in psoriasis (31, 46).

Leeuwen-Kerkhoff et al. and Cros et al. reported a low IL-12 production and Th1 programming capacity of slanMo (11, 42). In these studies, the short maturation step through which slanMo gain their outstanding IL-12 and IL-23 producing capacity was not taken into account. In addition, some groups rely on staining of CD16 in addition to slan for the isolation of slanMo. However, CD16 cross-linking can induce an inhibitory signal (inhibitory ITAM signaling, ITAMi), reducing pro-inflammatory cytokine production (58, 59). In addition, we realized that slanMo are sensitive to flow cytometric cell sorting as they rapidly undergo apoptosis thereafter. In summary, studies revealed that slanMo circulate in blood as immature cells that readily produce TNF-α and acquire the capacity to produce IL-12 and IL-23.

## Immune Cross-Talk of slanMo With Other Cells

### T Lymphocytes

Mononuclear phagocytes largely differ in their function to regulate adaptive immune responses by directing the quality and magnitude of T cell responses (Figure 1). Different studies assessed the function of slanMo to stimulate T cell proliferation and direct the production of T cell derived cytokines (16, 31, 60–62). slanMo revealed a better capacity to stimulate the proliferation of allogeneic CD4^+^ T cells than CD14^+^ monocytes (16, 26, 31). In contrast to CD14^+^ monocytes, slanMo efficiently primed T cells for the neoantigen keyhole limpet hemocyanin (KLH), and induced allo-antigen specific CD8^+^ cytotoxic T cells (33). Similar to CD1c^+^ DCs (DC2), slanMo primed naïve allogenic cord blood T cells (26). Further, slanMo demonstrated a stronger programming of Th1 cells as compared to DC2 when cultured for 6 h before stimulation with LPS and then co-culture with cord blood T cells (26, 45). This is in line with the superior IL-12 production of slanMo when compared with DC2 after 6 h of spontaneous maturation (54). Another study assessed the Th17 programming capacity using allogenic naïve T cells. Here again a higher capacity of slanMo to induce Th17/Th1 T cells was observed in cultures stimulated with slanMo instead of DC2 (31). The strong T cell stimulatory capacity of slanMo may be relevant for recall responses in peripheral tissues as well as for the priming of naïve T cells in lymphoid tissue (46).

### Natural Killer Cells

The interaction of mononuclear phagocytes and natural killer (NK) cells is well known. The main mechanisms by which mononuclear phagocytes can activate NK cells are soluble mediators as well as through direct cell-to-cell contacts. Co-culture of slanMo with NK cells promotes mutual activation (63, 64). Stimulation of slanMo with LPS induced an IL-12 production that stimulated NK cells to produce IFN-γ, which in a positive forward feedback loop potentiated the IL-12 production of slanMo and the IFN-γ production of NK cells. This resulted in an increased NK cell activation (CD69, NKp30, NKp44, NKG2D) as well as an increased tumor-directed cytotoxicity against chronic myeloid leukemia (CML) blasts and the leukemia cell line K562 compared to those NK cells stimulated without slanMo (63, 65). This cross talk of slanMo and NK cells also improved the slanMo-mediated differentiation of näive CD4^+^ T cells into IFN-γ producing Th1 cells (66).

Optimal reciprocal activation of slanMo and NK cells required direct cellular contact. Tufa et al. identified a cellular communication circuit through transmembrane TNF-α expressed by slanMo and its interaction with upregulated TNFR2 on NK cells leading to higher secretion of GM-CSF by NK cells (65). Similarly, ICAM-1 expressed by slanMo bound to LFA-1 on NK cells thereby promoting an enhanced IL-1β secretion by slanMo (63). Stimulation of slanMo with TLR7/8 ligands resulted in a pronounced production of TNF-α, IL-1β, IL-12, and IL-6 allowing for an improved tumor directed cytotoxicity of slanMo and NK cells (67).

### Neutrophils

Human neutrophils were shown to directly interact with both NK cells and slanMo *in vitro*, which eventually enhanced the activity of both cell types—NK cells and slanMo—after LPS, IL-12 or IL-12/IL-18 stimulation (64). Neutrophils engaged with slanMo via CD18 (integrin ß2) and intracellular adhesion molecule 1 (ICAM-1) that boosted the release of IL-12 by slanMo, which further stimulated activated NK cells to produce IFN-γ. Neutrophils were also shown to interact with NK cells via CD18 and ICAM-3 thereby augmenting IFN-γ production by NK cells (68). Co-localization of slanMo, NK cell, and neutrophils in inflamed tissue of psoriasis and Crohn's disease provided evidence for cooperation between these cells in which neutrophils may function as amplifiers of immune responses mediated by slanMo and NK cells.

### Mesenchymal Stem Cells

Mesenchymal stem cells (MSCs) are well known for their immunomodulatory properties. Results from therapeutic studies are encouraging and there is hope that the application of MSCs open new options for the therapy of immune-related diseases (69). slanMo were found in increased numbers in tissues affected by chronic inflammatory diseases such as Crohn's disease, multiple sclerosis, rheumatoid arthritis, and lupus erythematosus. Treatment with MSCs was regarded successful in these diseases. Co-culture of slanMo with MSCs resulted in a reduced production of TNF-α, IL-6, and IL-12, while production of the immunosuppressive cytokine IL-10 was enhanced in response to LPS stimulation (70). MSCs also inhibited slanMo-induced proliferation of allogeneic CD4^+^ and CD8^+^ T cells and dampened the polarization of naïve CD4^+^ T lymphocytes into Th1 cells (70). In these experiments prostaglandin E2 (PGE2) was identified as a main MSC-derived immune regulatory molecule. These findings fit well with the overall function of MSCs. Other MSC-derived immunoregulatory molecules are IL-10, IL-4, TGF-β, HGF, and PDL-1, all of which act by inhibiting differentiation of autoreactive CD4^+^ T cells into pathogenic Th1 cells by stimulating their differentiation into Th2 and Treg lymphocytes (69). These data suggest that MSCs considerably impair the immunostimulatory properties of inflammatory slanMo.

## slanMo *in vivo*

In healthy individuals, roughly 1% of PBMCs stain positive for the slan marker (71). In healthy stem cell donors treated with granulocyte-colony stimulating factor (G-CSF), the frequency of slanMo increased from 14.9 × 10^6^/L to 64.0 × 10^6^/L. G-CSF was described to increase the numbers of tolerogenic DCs and T cells among mobilized blood leukocytes in the graft (72). In contrast, slanMo mobilized by G-CSF retained their capacity to produce IL-12 and TNF-α (73). Furthermore, G-CSF–mobilized slanMo programmed the differentiation of Th1 cells and displayed a strong capacity to stimulate the proliferation of naïve allogeneic cord blood T cells (73). Thus, slanMo transfused into recipients of allogeneic peripheral blood stem cell (PBSC) transplants are functionally fully capable and may support graft-vs. -host disease as well as graft- vs. -leukemia effects.

During the first month after allogeneic stem cell transplantation slanMo showed slow reconstitution in blood compared to cDCs and pDCs (74), however, a steady increase in the frequency of slanMo has been observed in the 2nd and 3rd month after post-transplantation (75, 76). The slow reconstitution of slanMo after bone marrow transplantation as observed in this study is reflected by reports on non-classical monocytes demonstrating the same slow reconstitution in blood (18). Whether these findings argue for slanMo to develop from classical monocytes, as described for non-classical monocytes has not been addressed and requires further studies.

## slanMo in Diseases

The contribution of slanMo to the immune pathogenesis of different diseases has been studied (Table 2) and will be discussed in the following chapter.

**Table 2:** The observed location and potential role of slanMo in different diseases.

**Diseases**	**Presence**	**Function**	**Ref**.
Psoriasis	Higher frequency in skin lesions	Local expression of TNF-α, iNOS and IL-23	(31)
Atopic dermatitis	Higher frequency in skin lesions	Highly responsive to TLR4 or TLR7/8 ligands	(54)
Lupus nephritis(type III)	Selective accumulation in glomeruli with immune complex deposition	Local secretion of TNF-α and activation of endothelial cells	(43)
Lupus erythematosus	Higher frequency in skin lesions	Highly responsive to TLR7/TLR8 stimulation	(46)
Multiple sclerosis	Accumulation in highly inflamatory brain lesions	Local expression of TNF-α	(77)
Crohn 's disease	Abundent in inflamed ilial mucosa and mesentric lymphnodes	Local secretion of TNF-α and IL-1β	(78)
HIV	Higher frequency detected in peripheral blood	Secretion of TNF-α and IL-1β in peripheral blood	(71, 79)
Carcinoma	Presence in metastatic tumor draining lymphnodes	Efficient phagocytosis of tumor cells	(30)
Renal cell carcinoma	Increased frequency in ccRCC tissues	Higher frequency of slanMo associated with poor prognosis of ccRCC patients	(45)
Diffuse large B- cell lymphoma	Increased frequency in peripheral blood	Effector of antibody mediated cellular cytotoxicity	(29)

*iNOS- Inducible nitric oxide synthase, TLR- Toll-like receptors. ccRCC- clear cell renal cell carcinoma Ref.- References*.

### Psoriasis

Psoriasis is a chronic inflammatory skin disease with an immune response steered by IL-23 and TNF-α producing antigen presenting cells (16, 31, 80–82), thereby stimulating T cells to produce IL-17, a cytokine that is now identified to be of chief importance for inducing skin inflammation in psoriasis (83). Therapeutic responses to antibody mediated neutralization of IL-17, IL-23, and TNF-α (84, 85) underscore the role of these cytokines as predominant drivers of the disease.

slanMo have been found at increased frequencies in psoriasis skin lesions and these numbers rapidly normalized with clinically effective anti-TNF therapy (31, 84, 85). In parallel to the reduced numbers of slanMo in skin lesions their frequency in blood increased. Interestingly, these cells showed a decrease in their expression of HLA-DR (76, 85). Lesional slanMo expressed IL-23, TNF-α as well as inducible nitric oxide synthase (iNOS). The phenotype (CD1c^−^ and CD11c^+^) and function (IL23^+^, TNF-α^+^, iNOS^+^) of slanMo in active psoriasis skin lesions corresponded to TNF-α-producing iNOS expressing (TIP)-DCs, that were earlier defined by Lowes et al. (82). *In vitro* slanMo demonstrated the capacity to program T cells producing IFN-γ, IL-17, IL-22 but not IL-10 (16, 31, 81). These data lend additional support to the role of slanMo as relevant stimulatory cells in psoriasis.

Autocrine TNF-α stimulation of slanMo allows for high level production of IL-12, IL-23, IL-1ß, and IL-6 (56). In accord with the general role of TNF-α as a stimulatory cytokine, treatment with the potent TNF-α-inhibitor infliximab rapidly reduced IL-12, IL-1β, and CCL20 mRNA expression in psoriasis patients (84). The migration of slanMo from the peripheral blood into psoriasis skin lesions may be facilitated by the local expression of the anaphylatoxin C5a, fractalkine (CX3CL1), and CXCL12 for which the respective receptors are expressed by slanMo (C5aR, CX3CR1, and CXCR4) (31). Self-nucleic acid complexed to the antimicrobial peptide cathelicidin (LL37) is the best-studied autologous immune stimulus in psoriasis. Stimulating slanMo with LL37-RNA-complexes induced TNF-α production at higher levels compared to DC2 (31). The cytokine production clearly places these cells on center stage for orchestrating Th17-mediated immune responses in psoriasis. As there are other slan-negative antigen presenting cells producing IL-23 and TNF-α in psoriasis skin lesions, it remains to be elucidated whether slanMo have a unique and non-redundant stimulatory role in psoriasis skin inflammation. Given the selective expression of the slan on a pro-inflammatory cell type in psoriasis and other diseases, an antibody-directed targeting approach of slanMo has been developed (29, 31, 81, 86) that may have potential of serving as a new treatment option in psoriasis and other inflammatory diseases.

### Atopic Dermatitis

Atopic dermatitis (AD) is a chronic relapsing inflammatory skin disease affecting 15–25% of children and 1–3% of adults (87, 88). The changes within the mononuclear phagocyte system in AD are complex. Inflammatory epidermal dendritic cells (IDECs) (CD1a^+^, Langerin^−^, FcεRI^+^) are believed to enhance local inflammation and eczema severity in AD patients (89). Higher frequencies of dermal mononuclear phagocytes expressing CD11c, CD1a, CD206, and DC-SIGN have been identified in AD patients (90). Similar to psoriasis, a higher frequency of slanMo is also reported in the dermis of active skin lesions of AD patients. These slanMo lacked expression of FcεRI, CD1a, CD14, and CD163, thereby displaying a phenotype different from already described mononuclear phagocytes in AD patients (54). Peripheral blood slanMo of these patients retained their capacity to produce inflammatory cytokines and produced more TNF-α and IL-12 than myeloid DCs or classical monocytes after LPS or R848 stimulation (54). Mental stress is a well-known factor to trigger flares of AD (91). A standardized mental stress test in patients with AD induced an instant mobilization of slanMo into the blood circulation. Testing for their TNF-α-production showed their unchanged capacity to do so (54). The mobilization of CD16^+^ monocytes was previously shown for psoriasis patients (92). Whether this mobilization includes all CD16^+^ monocytes or applies preferentially to slanMo has not been addressed. Non-classical monocytes are known to function as patrolling monocytes along endothelial cells. Therefore, the observed stress induced mobilization may reflect detachment of slanMo from the vasculature into blood circulation. This process was shown to be induced by a transient rise of catecholamines induced by mental stress (54).

Cytokine production of slanMo is not a fixed condition and can be modulated by micro environmental factors relevant to AD and allergic diseases. Histamine is an important regulator of allergic inflammation that modulates pro-inflammatory functions of slanMo. Different histamine receptors are expressed by slanMo, particularly the recently identified histamine H4 receptor (H4R). Histamine effectively blunted TNF-α and IL-12 production of slanMo, a reduction mediated via the H4R and the combined action of H2R and H4R (93). Hence, H4R agonists might have therapeutic potential to down-regulate immune reactions, e.g., in allergic inflammatory skin diseases (93, 94). Birch pollen contains antigens potentially inducing allergic IgE-mediated sensitization. Pollen also contain immunomodulatory substances. In this context, pollen-associated E1-phytoprostanes (PPE1) were shown to license human monocyte-derived dendritic cell for T-helper type 2 (Th2) polarization of naïve T cells (95). Aqueous birch pollen extracts inhibited IL-12 production by slanMo in a dose-dependent manner, while the levels of IL-6 remained unaffected. PPE1 inhibited secretion of both IL-12p70 and IL-6. slanMo exposed to aqueous pollen extracts were impaired in eliciting an IFN-γ response in naïve CD4^+^ T cells (95). These data demonstrated that slanMo having a constitutively high potency to induce Th1 responses, are susceptible to the Th2 polarizing effect of low molecular weight, non-protein factors derived from pollen.

### Lupus Erythematosus

Lupus erythematosus is an autoimmune disease in which genetic and environmental factors lead to autoantibody production and induction of inflammation manifesting to multiple organs (96). The autoantibodies in lupus erythematosus patients are directed against nuclear antigens and form immune complexes containing double-stranded DNA (dsDNA) and single-stranded RNA (ssRNA) that activate DCs and drive pathogenic T cell responses (97–99). In response to ssRNA and dsDNA, plasmacytoid dendritic cells (pDCs) produce IFN-α, a critical immunoregulatory cytokine in lupus erythematosus (100, 101). slanMo were shown to lack IFN-α production but may contribute to the disease progression through high TNF-α production (46). Immunohistochemistry showed an increased frequency of slanMo in skin lesions of patients with cutaneous and systemic lupus erythematosus (46). slanMo were found scattered in the dermis where they locally expressed TNF-α. They appeared to cluster in lymph follicle-like structures where they co-localized with T cells. Incubating slanMo with serum from lupus erythematosus patients induced production of TNF-α (46). The stimulatory components of the lupus erythematosus sera are autoimmune complexes containing single-stranded RNA (ssRNA) binding to TLR7 and TLR8 or double stranded DNA binding to TLR9 (11). slanMo lack the DNA-sensor TLR9, but instead express TLR7 and TLR8 (46). In fact, ssRNA or selective TLR7 and TLR8 ligands induce TNF-α and IL-12 production in slanMo at higher levels compared to conventional dendritic cells (cDCs) or plasmacytoid dendritic cells (pDCs) (26, 31, 54).

Immune complexes binding to the vasculature frequently causes vasculitis in lupus patients (102, 103). In lupus nephritis, intracapillary accumulation of immune complexes can prime the activation of Fc receptor-bearing myeloid cells (99, 104, 105). The observation that slanMo have a CD16-mediated capacity to bind IgG-ICs (34) and to be present in lupus skin lesions (46) made us to investigate the role of IgG-ICs for the direct recruitment and activation of slanMo from the blood flow in lupus nephritis (34). Among the different types of lupus nephritis, intracapillary IC deposition and accumulation of monocytes are hallmarks of diffuse proliferative lupus nephritis class III and IV frequently leading to end stage renal disease (22, 106). The relevance of intracapillary IgG-ICs in terms of monocytes recruitment and activation, as well as the nature and function of these monocytes were not well understood. For the early focal form of lupus nephritis (class III) we demonstrated a selective accumulation of slanMo, which locally expressed TNF-α (43). *In vitro* and *in vivo* mouse studies showed that immobilized IgG-ICs induced a direct recruitment of slanMo from the microcirculation via interaction with FcγRIIIA (CD16) (43). Intravenous immunoglobulins block CD16 and completely prevented slanMo recruitment (34). Engagement of immobilized IgG-ICs by slanMo induced the production of neutrophil-attracting chemokine CXCL2 as well as TNF-α, which in a forward feedback loop stimulated endothelial cells to produce the slanMo-recruiting chemokine CX3CL1 (fractalkine) (43). These studies demonstrated that expression of CD16 equips slanMo with a capacity to orchestrate early IC-induced inflammatory responses in glomeruli and identified slanMo as a pathogenic cell type in lupus nephritis.

### Multiple Sclerosis (MS)

Multiple sclerosis is a chronic inflammatory disease of the central nervous system characterized by injury to the myelin sheath and axonal loss (107). Discussions of MS pathophysiology frequently put cells of the adaptive immune response in the spotlight. However, dendritic cells, monocytes, macrophages, and microglia, collectively referred to as mononuclear phagocytes, appear to have prominent roles in MS pathogenesis. These populations of mononuclear phagocytes function as antigen presenting effector cells in neuroinflammation (108–110). In a study on MS, slanMo were found in the patient's cerebrospinal fluid and accumulated in inflammatory brain lesions. The degree of local inflammation positively correlated with the number of slanMo (77). Recruitment of CXCR4 expressing slanMo to brain lesions may be induced by CXCL12, which was found elevated in MS patients (111, 112).

### Crohn's Disease

Crohn's disease is characterized by patchy inflammatory lesions and affects the entire gastrointestinal tract (113, 114). In humans, intestinal lamina propria, a subset of myeloid cells HLA-DR^high^ Lin^−^ CD14^+^ CD163^low^, have been identified that can enhance immunity and differentiation of Th17 cells (62). A study on slanMo revealed an increased frequency of IL-1β and TNF-α-producing slanMo in the mesenteric lymph nodes of Crohn's disease patients. slanMo accumulated in inflamed colons of Crohn's disease but not in ulcerative colitis patients (78). In parallel to the presence of slanMo in peripheral tissues, their frequency in blood circulation was reduced. Thus, slanMo may contribute to the immunopathogenesis of Crohn's disease.

### HIV Infection

Chronic immune activation and a breakdown of the gastrointestinal mucosal barrier allow translocation of microbial products (e.g., LPS) from gut associated lymphoid tissue into the circulation (115). LPS activates monocytes and DCs that produced pro-inflammatory cytokines such as TNF-α and IL-1β (71). Increased serum TNF-α has been reported for HIV-infected individuals and is known to promote viral replication in infected CD4^+^ T lymphocytes (116, 117). Therefore, the potential role of slanMo in fueling chronic immune activation during HIV-1 infection has been evaluated (71, 79). Dutertre et al. investigated the role of slanMo (referred to as mAb M-DC8^+^ monocytes) in peripheral blood of HIV infected individuals (79). Specifically, they addressed chronic immune hyperactivation caused by production of TNF-α. Viremic HIV patients showed an increase in CD16^+^ monocytes and a marked increase in slanMo (M-DC8^+^ cells). PBMCs of viremic patients displayed an overproduction of TNF-α in response to LPS that was mostly attributed to slanMo (79). Tufa et al. reported higher relative and absolute numbers of slanMo in peripheral blood of untreated HIV infected individuals, which were activated and secrete increased amounts of IL-1β, TNF-α and IL-12 compared to healthy controls. Furthermore, the frequency of IL-1β^+^ slanMo directly correlated with TNF-α^+^ slanMo and viral load, suggesting virus-driven immune activation of slanMo in HIV-infected individuals (71). These data are in support of a role of slanMo in the maintenance of chronic immune activation and HIV disease progression.

## slanMo in Cancer

Recently, slanMo have been implicated in a novel type of immune surveillance in cancer (45). Vermi et al. demonstrated that slanMo are recruited to metastatic tumor-draining lymph nodes (M-TDLN) where they are aligned along the tumor tissue (30). The recruitment of slanMo depended on the arrival of cancer cells to M-TDLN, as slanMo were absent in unaffected lymphnodes and at primary carcinoma sites. Within M-TDLN, slanMo were found adjacent to dead cells where they phagocytosed tumor cells (30). These slanMo expressed HLA-DR, CD40 and TNF-α. More importantly, unlike pDCs from the same patient cohort, circulating slanMo from patients with advanced colorectal cancer remained substantially intact in terms of numbers, cytokine production (TNF-α and IL-12p70) and induction of T-cell proliferation (62). Thus, in contrast to other mononuclear phagocytes these data suggested that circulating slanMo are not developmentally or functionally hijacked or converted into immunosuppressive cells by growing tumors.

A study on diffuse large B cell lymphoma highlighted slanMo as prominent effectors of antibody-mediated tumor cell targeting (29). slanMo from these patients showed an effective rituximab-mediated antibody dependent cell-mediated cellular cytotoxicity (ADCC) slightly lower when compared with the one displayed by NK cells. Moreover, slanMo acquired a macrophage-like phenotype and became very efficient in rituximab-mediated antibody dependent cellular phagocytosis (29). Previous studies identified the critical role of CD16 in slanMo mediated ADCC (118).

In multiple myeloma, numbers of circulating slanMo significantly reduced compared to healthy controls (119). Stimulation of bone marrow or peripheral blood from multiple myeloma patients with TLR7/8 ligand (R848) showed a reduced IL-12 production by slanMo. Further co-culture of slanMo with a multiple myeloma cell line or cells isolated from patients revealed a phenotypic shift of slanMo toward intermediate monocytes and these cells demonstrated a reduced capacity to induce T cell immune responses (119). In the tumor tissue of renal cell carcinoma, an increased number of slanMo have been reported, where they produced IL-10 and revealed a macrophage like phenotype (45).

Taken together, these studies identify different roles for slanMo in cancer. slanMo may be helpful by stimulating tumor specific T cells responses (32) and by conducting a tumor-directed cytotoxicity (ADCC) (29). On the other hand, slanMo can differentiate into cells that are part of a tolerogenic immune response (29, 30, 46). Thus, in cancer slanMo seem to display a remarkable functional plasticity.

## Controlling the Pro-Inflammatory Function of slanMo

In this chapter, we discuss studies investigating how the immune related function of slanMo is modulated by several common therapeutics that are applied for the treatment of chronic inflammatory diseases or cancer.

### PDE4-Inhibitor

A new option for the treatment of psoriasis and psoriasis arthritis is the phosphodiesterase 4 (PDE4)-inhibitor apremilast. PDE4-inhibitors increase intracellular cAMP levels and were shown to attenuate pro-inflammatory functions in different cell types and diseases (120, 121). Apremilast is currently tested in a phase III trial in Behçet's disease and is under study in a number of other inflammatory diseases (122, 123). Previous studies demonstrated that apremilast could reduce the production of GM-CSF, IL-12p70, TNF-α, and IFN-γ while increasing the production of IL-10 and IL-6 in LPS-stimulated PBMCs (124). Studies on ultraviolet B-irradiated keratinocytes showed a reduced production of TNF-α when cultured in the presence of apremilast while skin fibroblasts exhibited a reduced migratory capacity (125). Inhibition of PDE4 in slanMo reduced IL-12 and TNF-α production while this treatment enhanced their IL-23 production. As a consequence, apremilast-treated slanMo showed a reduced induction of Th1 cells while at the same time sustaining Th17 responses. A strong Th17-promoting effect of a drug that is effective in the treatment of an IL-17-mediated disease is unexpected. The enhanced IL-23p19 production in response to PDE4-inhibition can be explained by cAMP-dependent activation of protein kinase A and subsequent phosphorylation of the cAMP-response element binding protein (CREB) (126). Recently, the PDE4 inhibitor roflumilast licensed for the treatment of COPD was studied in mouse DCs generated *in vitro* from bone marrow precursor cells (127). In line with our study, these authors also demonstrated a PDE-4-inhibitor induced production of IL-23 in DCs and of IL-17 in T cells. Therefore, PDE4-inhibitors possibly exert their good therapeutic effects through modulation of functions on other immune and non-immune cells.

### Dimethylfumarate

Dimethylfumarate (DMF) is a small molecule licensed for the treatment of psoriasis and multiple sclerosis (128). Skin lesions of psoriasis patients treated with DMF (in combination with monomethylfumarate—fumaderm®) showed a reduced frequency of slanMo. Studying the function of slanMo in the presence of DMF demonstrated an inhibition of CX3CL1- and C5a-induced migration of slanMo. Both, CX3CL1- as well as C5a are expressed in psoriasis plaques. DMF also attenuated the rapid spontaneous phenotypic maturation of slanMo, as judged by reduced expression of CD80, CD86, CD83, and HLA-DR (124). In addition, slanMo showed a DMF-dependent decrease in the production of IL-23, IL-12, TNF-α, and IL-10, and a reduced capacity to stimulate Th17/Th1 responses. At the level of intracellular signaling, DMF-treated slanMo showed an increased expression of heme oxygenase 1 (HO-1) (124). HO-1 is an enzyme with import antioxidant, anti-inflammatory, and cytoprotective functions. Treatment of slanMo with DMF also inhibited phosphorylation of NFκB p65. This may directly affect IL-12p70 transcription as NFκB p65 binding sites were found within the IL-23p19 promoter (124). Moreover, the observed DMF-dependent reduction in STAT1 phosphorylation would explain the reduced IL-12/IL-23 production of DMF-treated slanMo, as STAT1 signaling is essential in this respect (124, 129). Collectively, these findings demonstrated that slanMo found in psoriasis as well as in MS are a relevant target for the therapeutic immunomodulatory effects of DMF.

### Chemotherapeutic Agent

Treatment of cancer with chemotherapeutic agents remains a challenge for immunological researcher. An ideal therapy should target the proliferation of cancer cells and leave the function of tumor-directed immune responses intact. In a study, comparing different chemotherapeutic agents for their *in vitro* capacity to modulate the function of slanMo, mitomycin-c, methotrexate, and paclitaxel have no influence on the ability of slanMo to secrete pro-inflammatory cytokines. The ability of treated slanMo to activate T lymphocytes and NK cells also remained intact (130). These observations provided arguments of slanMo contributing to tumor cell elimination in patients treated with respective drugs. However, in this context, doxorubicin and vinblastine significantly impaired production of TNF-α, IL-12, and IL-6 by slanMo (130). Both drugs also inhibited slanMo-mediated T cell proliferation and suppressed their ability to stimulate NK cells.

Bortezomib is an efficient targeted form of chemotherapy for treatment of multiple myeloma (131). The anti-tumor activity of bortezomib is mediated by proteasome inhibition, leading to NFκB inhibition, decreased cell proliferation and induction of apoptosis (132). Bortezomib mediated proteasome inhibition efficiently impaired *in vitro* maturation of slanMo as well as release of TNF-α and IL-12 upon LPS stimulation. In addition, it also inhibited slanMo-mediated proliferation and differentiation of CD4^+^ T cells. Furthermore, bortezomib impaired the ability of slanMo to stimulate IFN-γ secretion and tumor-directed cytotoxicity of NK cells (133).

## Conclusions and Perspectives

Many studies have contributed to the current understanding of slanMo as cells with a pronounced potential to stimulate innate and adaptive immune responses. slanMo appear similar but not identical to non-classical monocytes with some genes such as CXCR6 being differentially expressed. In the case of the most obvious difference, namely the selective expression of the slan-antigen, the function remains to be determined. Other open questions regard the precursor cells of slanMo and the signals guiding their development.

In regard to their likely function as patrolling monocytes, slanMo may play an unexplored role in immune surveillance of the vasculature. Our recent study in immune complex induced lupus nephritis would be in line with this task. Here slanMo were shown to play an important role for the initiation of the immune complex induced immune response in the glomerular capillaries. Whether this early stimulatory function during the beginning of an immune response holds true also for other inflammatory diseases such as psoriasis is an open question. Additional investigations to the function of slanMo in cancer are to be awaited and appear relevant as *in vitro* studies demonstrated their effective tumor-directed cytotoxicity and stimulation of tumor-directed immune responses.

Overall, there is a high interest in gaining a better understanding of the function of slanMo and slan-negative non-classical monocytes. Further in-depth studies of slanMo can be highly informative for understanding immunopathology and provide an attractive target for therapeutic intervention.

## Author Contributions

FA, KS, MS, and TD have written and edited the manuscript.

### Conflict of Interest Statement

The authors declare that the research was conducted in the absence of any commercial or financial relationships that could be construed as a potential conflict of interest.

## Abbreviations

PSGL1: P selectin glycoprotein ligand 1
slanMo: 6-sulfo LacNAc expressing monocytes
DCs: Dendritic cells
Ly: Lymphocyte antigen
ROS: Reactive oxygen species
HO-1: Heme oxygenase 1
PD-L1: Programmed death-ligand 1
TLR: Toll like receptor
NR4A1: Nuclear receptor transcription factor 4A1
G-CSF: Granulocyte-colony stimulating factor
LPS: Lipopolysaccharides
PBMCs: Peripheral blood mononuclear cells
COPD: Chronic Obstructive Pulmonary Diseases
HGF: Hepatocyte Growth Factor.

## References

[1] Guilliams M Mildner A Yona S. Developmental and functional heterogeneity of monocytes. Immunity. (2018) 49:595–613. 10.1016/j.immuni.2018.10.005 30332628

[2] Zhao Y Zou W Du J Zhao Y. The origins and homeostasis of monocytes and tissue-resident macrophages in physiological situation. J Cell Physiol. (2018) 233:6425–39. 10.1002/jcp.26461 29323706

[3] Passlick B Flieger D Ziegler-Heitbrock HW. Identification and characterization of a novel monocyte subpopulation in human peripheral blood. Blood. (1989) 74:2527–34. 2478233

[4] Ziegler-Heitbrock L Ancuta P Crowe S Dalod M Grau V Hart DN. Nomenclature of monocytes and dendritic cells in blood. Blood. (2010) 116:e74-80. 10.1182/blood-2010-02-258558 20628149

[5] Auffray C Sieweke MH Geissmann F. Blood monocytes: development, heterogeneity, and relationship with dendritic cells. Annu Rev Immunol. (2009) 27:669–92. 10.1146/annurev.immunol.021908.132557 19132917

[6] Ingersoll MA Spanbroek R Lottaz C Gautier EL Frankenberger M Hoffmann R. Comparison of gene expression profiles between human and mouse monocyte subsets. Blood. (2010) 115:e10-9. 10.1182/blood-2009-07-235028 19965649 PMC2810986

[7] Imhof BA Jemelin S Ballet R Vesin C Schapira M Karaca M. CCN1/CYR61-mediated meticulous patrolling by Ly6Clow monocytes fuels vascular inflammation. Proc Natl Acad Sci USA. (2016) 113:E4847–56. 10.1073/pnas.1607710113 27482114 PMC4995973

[8] Thomas G Tacke R Hedrick CC Hanna RN. Nonclassical patrolling monocyte function in the vasculature. Arterioscler Thromb Vasc Biol. (2015) 35:1306–16. 10.1161/ATVBAHA.114.304650 25838429 PMC4441550

[9] Auffray C Fogg D Garfa M Elain G Join-Lambert O Kayal S. Monitoring of blood vessels and tissues by a population of monocytes with patrolling behavior. Science. (2007) 317:666–70. 10.1126/science.1142883 17673663

[10] Hanna RN Cekic C Sag D Tacke R Thomas GD Nowyhed H. Patrolling monocytes control tumor metastasis to the lung. Science. (2015) 350:985–90. 10.1126/science.aac9407 26494174 PMC4869713

[11] Cros J Cagnard N Woollard K Patey N Zhang SY Senechal B. Human CD14dim monocytes patrol and sense nucleic acids and viruses via TLR7 and TLR8 receptors. Immunity. (2010) 33:375–86. 10.1016/j.immuni.2010.08.012 20832340 PMC3063338

[12] Buscher K Marcovecchio P Hedrick CC Ley K. Patrolling mechanics of non-classical monocytes in vascular inflammation. Front Cardiovasc Med. (2017) 4:80. 10.3389/fcvm.2017.00080 29312957 PMC5742122

[13] Finsterbusch M Hall P Li A Devi S Westhorpe CL Kitching AR. Patrolling monocytes promote intravascular neutrophil activation and glomerular injury in the acutely inflamed glomerulus. Proc Natl Acad Sci USA. (2016) 113:E5172–81. 10.1073/pnas.1606253113 27528685 PMC5024581

[14] Anbazhagan K Duroux-Richard I Jorgensen C Apparailly F. Transcriptomic network support distinct roles of classical and non-classical monocytes in human. Int Rev Immunol. (2014) 33:470–89. 10.3109/08830185.2014.902453 24730730

[15] Stansfield BK Ingram DA. Clinical significance of monocyte heterogeneity. Clin Transl Med. (2015) 4:5. 10.1186/s40169-014-0040-3 25852821 PMC4384980

[16] Schakel K von Kietzell M Hansel A Ebling A Schulze L Haase M. Human 6-sulfo LacNAc-expressing dendritic cells are principal producers of early interleukin-12 and are controlled by erythrocytes. Immunity. (2006) 24:767–77. 10.1016/j.immuni.2006.03.020 16782032

[17] Hilgendorf I Gerhardt LM Tan TC Winter C Holderried TA Chousterman BG. Ly-6Chigh monocytes depend on Nr4a1 to balance both inflammatory and reparative phases in the infarcted myocardium. Circ Res. (2014) 114:1611–22. 10.1161/CIRCRESAHA.114.303204 24625784 PMC4017349

[18] Patel AA Zhang Y Fullerton JN Boelen L Rongvaux A Maini AA. The fate and lifespan of human monocyte subsets in steady state and systemic inflammation. J Exp Med. (2017) 214:1913–23. 10.1084/jem.20170355 28606987 PMC5502436

[19] Hanna RN Carlin LM Hubbeling HG Nackiewicz D Green AM Punt JA. The transcription factor NR4A1 (Nur77) controls bone marrow differentiation and the survival of Ly6C- monocytes. Nat Immunol. (2011) 12:778–85. 10.1038/ni.2063 21725321 PMC3324395

[20] Carlin LM Stamatiades EG Auffray C Hanna RN Glover L Vizcay-Barrena G. Nr4a1-dependent Ly6C(low) monocytes monitor endothelial cells and orchestrate their disposal. Cell. (2013) 153:362–75. 10.1016/j.cell.2013.03.010 23582326 PMC3898614

[21] Zhong H Bao W Li X Miller A Seery C Haq N. CD16+ monocytes control T-cell subset development in immune thrombocytopenia. Blood. (2012) 120:3326–35. 10.1182/blood-2012-06-434605 22915651 PMC3476543

[22] Yoshimoto S Nakatani K Iwano M Asai O Samejima K Sakan H. Elevated levels of fractalkine expression and accumulation of CD16+ monocytes in glomeruli of active lupus nephritis. Am J Kidney Dis. (2007) 50:47–58. 10.1053/j.ajkd.2007.04.012 17591524

[23] Wong KL Yeap WH Tai JJ Ong SM Dang TM Wong SC. The three human monocyte subsets: implications for health and disease. Immunol Res. (2012) 53:41–57. 10.1007/s12026-012-8297-3 22430559

[24] Kowal K Moniuszko M Dabrowska M Bodzenta-Lukaszyk A. Allergen challenge differentially affects the number of circulating monocyte subsets. Scand J Immunol. (2012) 75:531–9. 10.1111/j.1365-3083.2012.02685.x 22260220

[25] Ziegler-Heitbrock L Hofer TP. Toward a refined definition of monocyte subsets. Front Immunol. (2013) 4:23. 10.3389/fimmu.2013.00023 23382732 PMC3562996

[26] Schakel K Kannagi R Kniep B Goto Y Mitsuoka C Zwirner J. 6-Sulfo LacNAc, a novel carbohydrate modification of PSGL-1, defines an inflammatory type of human dendritic cells. Immunity. (2002) 17:289–301. 10.1016/S1074-7613(02)00393-X 12354382

[27] Siedlar M Frankenberger M Ziegler-Heitbrock LH Belge KU. The M-DC8-positive leukocytes are a subpopulation of the CD14+ CD16+ monocytes. Immunobiology. (2000) 202:11–7. 10.1016/S0171-2985(00)80047-9 10879684

[28] Micheletti A Finotti G Calzetti F Lonardi S Zoratti E Bugatti M slan/M-DC8+ cells constitute a distinct subset of dendritic cells in human tonsils. Oncotarget. (2016) 7:161–75. 10.18632/oncotarget.6660 26695549 PMC4807990

[29] Vermi W Micheletti A Finotti G Tecchio C Calzetti F Costa S. slan+ monocytes and macrophages mediate CD20-dependent B cell lymphoma elimination via ADCC and ADCP. Cancer Res. (2018) 78:3544–59. 10.1158/0008-5472.CAN-17-2344 29748373

[30] Vermi W Micheletti A Lonardi S Costantini C Calzetti F Nascimbeni R. slanDCs selectively accumulate in carcinoma-draining lymph nodes and marginate metastatic cells. Nat Commun. (2014) 5:3029. 10.1038/ncomms4029 24398631

[31] Hansel A Gunther C Ingwersen J Starke J Schmitz M Bachmann M. Human slan (6-sulfo LacNAc) dendritic cells are inflammatory dermal dendritic cells in psoriasis and drive strong TH17/TH1 T-cell responses. J Allergy Clin Immunol. (2011) 127:787-94 e1-9. 10.1016/j.jaci.2010.12.009 21377044

[32] Schakel K Mayer E Federle C Schmitz M Riethmuller G Rieber EP. A novel dendritic cell population in human blood: one-step immunomagnetic isolation by a specific mAb (M-DC8) and *in vitro priming of cytotoxic T lymphocytes*. Eur J Immunol. (1998) 28:4084–93. 10.1002/(SICI)1521-4141(199812)28:12<4084::AID-IMMU4084>3.0.CO;2-4 9862344

[33] Schakel K Poppe C Mayer E Federle C Riethmuller G Rieber EP. M-DC8+ leukocytes–a novel human dendritic cell population. Pathobiology. (1999) 67:287–90. 10.1159/000028081 10725804

[34] Dobel T Kunze A Babatz J Trankner K Ludwig A Schmitz M. FcgammaRIII (CD16) equips immature 6-sulfo LacNAc-expressing dendritic cells (slanDCs) with a unique capacity to handle IgG-complexed antigens. Blood. (2013) 121:3609–18. 10.1182/blood-2012-08-447045 23460612

[35] Yu SY Hsiao CT Izawa M Yusa A Ishida H Nakamura S. Distinct substrate specificities of human GlcNAc-6-sulfotransferases revealed by mass spectrometry-based sulfoglycomic analysis. J Biol Chem. (2018) 293:15163–77. 10.1074/jbc.RA118.001937 30093410 PMC6166739

[36] Bochner BS Alvarez RA Mehta P Bovin NV Blixt O White JR. Glycan array screening reveals a candidate ligand for Siglec-8. J Biol Chem. (2005) 280:4307–12. 10.1074/jbc.M412378200 15563466

[37] Campanero-Rhodes MA Childs RA Kiso M Komba S Le Narvor C Warren J. Carbohydrate microarrays reveal sulphation as a modulator of siglec binding. Biochem Biophys Res Commun. (2006) 344:1141–6. 10.1016/j.bbrc.2006.03.223 16647038

[38] Carlsson S Oberg CT Carlsson MC Sundin A Nilsson UJ Smith D. Affinity of galectin-8 and its carbohydrate recognition domains for ligands in solution and at the cell surface. Glycobiology. (2007) 17:663–76. 10.1093/glycob/cwm026 17339281

[39] Tateno H Ohnishi K Yabe R Hayatsu N Sato T Takeya M. Dual specificity of Langerin to sulfated and mannosylated glycans via a single C-type carbohydrate recognition domain. J Biol Chem. (2010) 285:6390–400. 10.1074/jbc.M109.041863 20026605 PMC2825434

[40] Ideo H Matsuzaka T Nonaka T Seko A Yamashita K. Galectin-8-N-domain recognition mechanism for sialylated and sulfated glycans. J Biol Chem. (2011) 286:11346–55. 10.1074/jbc.M110.195925 21288902 PMC3064191

[41] Kletter D Singh S Bern M Haab BB. Global comparisons of lectin-glycan interactions using a database of analyzed glycan array data. Mol Cell Proteomics. (2013) 12:1026–35. 10.1074/mcp.M112.026641 23399549 PMC3617327

[42] van Leeuwen-Kerkhoff N Lundberg K Westers TM Kordasti S Bontkes HJ de Gruijl TD Transcriptional profiling reveals functional dichotomy between human slan(+) non-classical monocytes and myeloid dendritic cells. J Leukoc Biol. (2017) 102:1055–68. 10.1189/jlb.3MA0117-037R 28720687

[43] Olaru F Dobel T Lonsdorf AS Oehrl S Maas M Enk AH. Intracapillary immune complexes recruit and activate slan-expressing CD16+ monocytes in human lupus nephritis. JCI Insight. (2018) 3:96942. 10.1172/jci.insight.96492 29875315 PMC6124405

[44] Calzetti F Tamassia N Micheletti A Finotti G Bianchetto-Aguilera F Cassatella MA Human dendritic cell subset 4 (DC4) correlates to a subset of CD14(dim/-)CD16(++) monocytes. J Allergy Clin Immunol. (2018) 141:2276–9.e3. 10.1016/j.jaci.2017.12.988 29366702

[45] Toma M Wehner R Kloss A Hubner L Fodelianaki G Erdmann K. Accumulation of tolerogenic human 6-sulfo LacNAc dendritic cells in renal cell carcinoma is associated with poor prognosis. Oncoimmunology. (2015) 4:e1008342. 10.1080/2162402X.2015.1008342 26155414 PMC4485722

[46] Hansel A Gunther C Baran W Bidier M Lorenz HM Schmitz M. Human 6-sulfo LacNAc (slan) dendritic cells have molecular and functional features of an important pro-inflammatory cell type in lupus erythematosus. J Autoimmun. (2013) 40:1–8. 10.1016/j.jaut.2012.07.005 22890025

[47] Hamers AAJ Dinh HQ Thomas GD Marcovecchio P Blatchley A Nakao CS. Human monocyte heterogeneity as revealed by high-dimensional mass cytometry. Arterioscler Thromb Vasc Biol. (2019) 39:25–36. 10.1161/ATVBAHA.118.311022 30580568 PMC6697379

[48] Hofnagel O Engel T Severs NJ Robenek H Buers I. SR-PSOX at sites predisposed to atherosclerotic lesion formation mediates monocyte-endothelial cell adhesion. Atherosclerosis. (2011) 217:371–8. 10.1016/j.atherosclerosis.2011.04.021 21612780

[49] Gunther C Carballido-Perrig N Kaesler S Carballido JM Biedermann T. CXCL16 and CXCR6 are upregulated in psoriasis and mediate cutaneous recruitment of human CD8+ T cells. J Invest Dermatol. (2012) 132:626–34. 10.1038/jid.2011.371 22113484

[50] Qin M Guo Y Jiang L Wang X. Elevated levels of serum sCXCL16 in systemic lupus erythematosus; potential involvement in cutaneous and renal manifestations. Clin Rheumatol. (2014) 33:1595–601. 10.1007/s10067-014-2741-9 25015061

[51] Gutwein P Abdel-Bakky MS Schramme A Doberstein K Kampfer-Kolb N Amann K. CXCL16 is expressed in podocytes and acts as a scavenger receptor for oxidized low-density lipoprotein. Am J Pathol. (2009) 174:2061–72. 10.2353/ajpath.2009.080960 19435795 PMC2684172

[52] Dahl CP Husberg C Gullestad L Waehre A Damas JK Vinge LE. Increased production of CXCL16 in experimental and clinical heart failure: a possible role in extracellular matrix remodeling. Circ Heart Fail. (2009) 2:624–32. 10.1161/CIRCHEARTFAILURE.108.821074 19919988

[53] Hofer TP Zawada AM Frankenberger M Skokann K Satzl AA Gesierich W. slan-defined subsets of CD16-positive monocytes: impact of granulomatous inflammation and M-CSF receptor mutation. Blood. (2015) 126:2601–10. 10.1182/blood-2015-06-651331 26443621

[54] Baran W Oehrl S Ahmad F Dobel T Alt C Buske-Kirschbaum A. Phenotype, function, and mobilization of 6-sulfo LacNAc-expressing monocytes in atopic dermatitis. Front Immunol. (2018) 9:1352. 10.3389/fimmu.2018.01352 29977237 PMC6021776

[55] Schmitz M Zhao S Deuse Y Schakel K Wehner R Wohner H. Tumoricidal potential of native blood dendritic cells: direct tumor cell killing and activation of NK cell-mediated cytotoxicity. J Immunol. (2005) 174:4127–34. 10.4049/jimmunol.174.7.4127 15778372

[56] Kunze A Forster U Oehrl S Schmitz M Schakel K. Autocrine TNF-alpha and IL-1beta prime 6-sulfo LacNAc(+) dendritic cells for high-level production of IL-23. Exp Dermatol. (2017) 26:314–6. 10.1111/exd.13134 27315572

[57] Poltorak MP Zielinski CE. Hierarchical governance of cytokine production by 6-sulfo LacNAc (slan) dendritic cells for the control of psoriasis pathogenesis. Exp Dermatol. (2017) 26:317–8. 10.1111/exd.13170 27541644

[58] Aloulou M Ben Mkaddem S Biarnes-Pelicot M Boussetta T Souchet H Rossato E. IgG1 and IVIg induce inhibitory ITAM signaling through FcgammaRIII controlling inflammatory responses. Blood. (2012) 119:3084–96. 10.1182/blood-2011-08-376046 22337713

[59] Zarbock A Muller H Kuwano Y Ley K. PSGL-1-dependent myeloid leukocyte activation. J Leukoc Biol. (2009) 86:1119–24. 10.1189/jlb.0209117 19703898

[60] Oehrl S Prakash H Ebling A Trenkler N Wolbing P Kunze A. The phosphodiesterase 4 inhibitor apremilast inhibits Th1 but promotes Th17 responses induced by 6-sulfo LacNAc (slan) dendritic cells. J Dermatol Sci. (2017) 87:110–5. 10.1016/j.jdermsci.2017.04.005 28499587

[61] Kumar NA Cheong K Powell DR da Fonseca Pereira C Anderson J Evans VA. The role of antigen presenting cells in the induction of HIV-1 latency in resting CD4(+) T-cells. Retrovirology. (2015) 12:76. 10.1186/s12977-015-0204-2 26362311 PMC4567795

[62] Ogino T Nishimura J Barman S Kayama H Uematsu S Okuzaki D. Increased Th17-inducing activity of CD14+ CD163 low myeloid cells in intestinal lamina propria of patients with Crohn's disease. Gastroenterology. (2013) 145:1380–91 e1. 10.1053/j.gastro.2013.08.049 23993972

[63] Tufa DM Ahmad F Chatterjee D Ahrenstorf G Schmidt RE Jacobs R. IL-1beta limits the extent of human 6-sulfo LacNAc dendritic cell (slanDC)-mediated NK cell activation and regulates CD95-induced apoptosis. Cell Mol Immunol. (2017) 14:976–85. 10.1038/cmi.2016.17 27086951 PMC5719135

[64] Micheletti A Costantini C Calzetti F Camuesco D Costa S Tamassia N. Neutrophils promote 6-sulfo LacNAc+ dendritic cell (slanDC) survival. J Leukoc Biol. (2013) 94:705–10. 10.1189/jlb.1212638 23559493

[65] Tufa DM Chatterjee D Low HZ Schmidt RE Jacobs R. TNFR2 and IL-12 coactivation enables slanDCs to support NK-cell function via membrane-bound TNF-alpha. Eur J Immunol. (2014) 44:3717–28. 10.1002/eji.201444676 25229755

[66] Wehner R Lobel B Bornhauser M Schakel K Cartellieri M Bachmann M. Reciprocal activating interaction between 6-sulfo LacNAc+ dendritic cells and NK cells. Int J Cancer. (2009) 124:358–66. 10.1002/ijc.23962 18942710

[67] Jahnisch H Wehner R Tunger A Kunze A Oehrl S Schakel K. TLR7/8 agonists trigger immunostimulatory properties of human 6-sulfo LacNAc dendritic cells. Cancer Lett. (2013) 335:119–27. 10.1016/j.canlet.2013.02.003 23402811

[68] Costantini C Calzetti F Perbellini O Micheletti A Scarponi C Lonardi S. Human neutrophils interact with both 6-sulfo LacNAc+ DC and NK cells to amplify NK-derived IFN{gamma}: role of CD18, ICAM-1, and ICAM-3. Blood. (2011) 117:1677–86. 10.1182/blood-2010-06-287243 21098395

[69] Leyendecker A Jr Pinheiro CCG Amano MT Bueno DF. The use of human mesenchymal stem cells as therapeutic agents for the *in vivo* treatment of immune-related diseases: a systematic review. Front Immunol. (2018) 9:2056. 10.3389/fimmu.2018.02056 30254638 PMC6141714

[70] Wehner R Wehrum D Bornhauser M Zhao S Schakel K Bachmann MP Mesenchymal stem cells efficiently inhibit the pro-inflammatory properties of 6-sulfo LacNAc dendritic cells. Haematologica. (2009) 94:1151–6. 10.3324/haematol.2008.001735 19546436 PMC2719037

[71] Tufa DM Ahmad F Chatterjee D Ahrenstorf G Schmidt RE Jacobs R. Brief report: HIV-1 infection results in increased frequency of active and inflammatory SlanDCs that produce high level of IL-1beta. J Acquir Immune Defic Syndr. (2016) 73:34–8. 10.1097/QAI.0000000000001082 27243902

[72] Bensinger WI Weaver CH Appelbaum FR Rowley S Demirer T Sanders J. Transplantation of allogeneic peripheral blood stem cells mobilized by recombinant human granulocyte colony-stimulating factor. Blood. (1995) 85:1655–8. 7534140

[73] Baumeister SH Holig K Bornhauser M Meurer M Rieber EP Schakel K G-CSF mobilizes slanDCs (6-sulfo LacNAc+ dendritic cells) with a high pro-inflammatory capacity. Blood. (2007) 110:3078–81. 10.1182/blood-2006-12-062984 17616642

[74] Mimiola E Marini O Perbellini O Micheletti A Vermi W Lonardi S. Rapid reconstitution of functionally active 6-sulfoLacNAc(+) dendritic cells (slanDCs) of donor origin following allogeneic haematopoietic stem cell transplant. Clin Exp Immunol. (2014) 178:129–41. 10.1111/cei.12387 24853271 PMC4360203

[75] Mager K Wehner R Bahr F Oelschlagel U Platzbecker U Wermke M. Reconstitution of 6-sulfo LacNAc dendritic cells after allogeneic stem-cell transplantation. Transplantation. (2012) 93:1270–5. 10.1097/TP.0b013e31824fd8b4 22643330

[76] Baran W Koziol M Wozniak Z Banasik M Boratynska M Kunze A. Increased numbers of 6-sulfo LacNAc (slan) dendritic cells in hand transplant recipients. Ann Transplant. (2015) 20:649–54. 10.12659/AOT.894828 26510404

[77] Thomas K Dietze K Wehner R Metz I Tumani H Schultheiss T. Accumulation and therapeutic modulation of 6-sulfo LacNAc(+) dendritic cells in multiple sclerosis. Neurol Neuroimmunol Neuroinflamm. (2014) 1:e33. 10.1212/NXI.0000000000000033 25340085 PMC4204231

[78] Bsat M Chapuy L Baba N Rubio M Panzini B Wassef R Differential accumulation and function of pro-inflammatory 6-sulfo LacNAc dendritic cells in lymph node and colon of Crohn's versus ulcerative colitis patients. J Leukoc Biol. (2015) 98:671–81. 10.1189/jlb.5A1014-509RR 26162403

[79] Dutertre CA Amraoui S DeRosa A Jourdain JP Vimeux L Goguet M. Pivotal role of M-DC8(+) monocytes from viremic HIV-infected patients in TNFalpha overproduction in response to microbial products. Blood. (2012) 120:2259–68. 10.1182/blood-2012-03-418681 22802339

[80] Weaver CT Hatton RD Mangan PR Harrington LE. IL-17 family cytokines and the expanding diversity of effector T cell lineages. Annu Rev Immunol. (2007) 25:821–52. 10.1146/annurev.immunol.25.022106.141557 17201677

[81] Gunther C Starke J Zimmermann N Schakel K. Human 6-sulfo LacNAc (slan) dendritic cells are a major population of dermal dendritic cells in steady state and inflammation. Clin Exp Dermatol. (2012) 37:169–76. 10.1111/j.1365-2230.2011.04213.x 22188261

[82] Lowes MA Chamian F Abello MV Fuentes-Duculan J Lin SL Nussbaum R. Increase in TNF-alpha and inducible nitric oxide synthase-expressing dendritic cells in psoriasis and reduction with efalizumab (anti-CD11a). Proc Natl Acad Sci USA. (2005) 102:19057–62. 10.1073/pnas.0509736102 16380428 PMC1323218

[83] Brembilla NC Senra L Boehncke WH. The IL-17 family of cytokines in psoriasis: IL-17A and beyond. Front Immunol. (2018) 9:1682. 10.3389/fimmu.2018.01682 30127781 PMC6088173

[84] Brunner PM Koszik F Reininger B Kalb ML Bauer W Stingl G. Infliximab induces downregulation of the IL-12/IL-23 axis in 6-sulfo-LacNac (slan)+ dendritic cells and macrophages. J Allergy Clin Immunol. (2013) 132:1184–93 e8. 10.1016/j.jaci.2013.05.036 23890755

[85] Gunther C Blau K Forster U Viehweg A Wozel G Schakel K. Reduction of inflammatory slan (6-sulfo LacNAc) dendritic cells in psoriatic skin of patients treated with etanercept. Exp Dermatol. (2013) 22:535–40. 10.1111/exd.12190 23879812

[86] Bippes CC Feldmann A Stamova S Cartellieri M Schwarzer A Wehner R. A novel modular antigen delivery system for immuno targeting of human 6-sulfo LacNAc-positive blood dendritic cells (SlanDCs). PLoS ONE. (2011) 6:e16315. 10.1371/journal.pone.0016315 21283706 PMC3025022

[87] Leung DY Bieber T. Atopic dermatitis. Lancet. (2003) 361:151–60. 10.1016/S0140-6736(03)12193-9 12531593

[88] Werfel T Allam JP Biedermann T Eyerich K Gilles S Guttman-Yassky E. Cellular and molecular immunologic mechanisms in patients with atopic dermatitis. J Allergy Clin Immunol. (2016) 138:336–49. 10.1016/j.jaci.2016.06.010 27497276

[89] Yoshida K Kubo A Fujita H Yokouchi M Ishii K Kawasaki H. Distinct behavior of human Langerhans cells and inflammatory dendritic epidermal cells at tight junctions in patients with atopic dermatitis. J Allergy Clin Immunol. (2014) 134:856–64. 10.1016/j.jaci.2014.08.001 25282566

[90] Skabytska Y Wolbing F Gunther C Koberle M Kaesler S Chen KM. Cutaneous innate immune sensing of Toll-like receptor 2-6 ligands suppresses T cell immunity by inducing myeloid-derived suppressor cells. Immunity. (2014) 41:762–75. 10.1016/j.immuni.2014.10.009 25456159

[91] Arndt J Smith N Tausk F. Stress and atopic dermatitis. Curr Allergy Asthma Rep. (2008) 8:312–7. 10.1007/s11882-008-0050-6 18606083

[92] Buske-Kirschbaum A Kern S Ebrecht M Hellhammer DH. Altered distribution of leukocyte subsets and cytokine production in response to acute psychosocial stress in patients with psoriasis vulgaris. Brain Behav Immun. (2007) 21:92–9. 10.1016/j.bbi.2006.03.006 16714097

[93] Gschwandtner M Schakel K Werfel T Gutzmer R. Histamine H(4) receptor activation on human slan-dendritic cells down-regulates their pro-inflammatory capacity. Immunology. (2011) 132:49–56. 10.1111/j.1365-2567.2010.03336.x 20722760 PMC3015074

[94] Thangam EB Jemima EA Singh H Baig MS Khan M Mathias CB. The role of histamine and histamine receptors in mast cell-mediated allergy and inflammation: the hunt for new therapeutic targets. Front Immunol. (2018) 9:1873. 10.3389/fimmu.2018.01873 30150993 PMC6099187

[95] Gilles S Jacoby D Blume C Mueller MJ Jakob T Behrendt H. Pollen-derived low-molecular weight factors inhibit 6-sulfo LacNAc+ dendritic cells' capacity to induce T-helper type 1 responses. Clin Exp Allergy. (2010) 40:269–78. 10.1111/j.1365-2222.2009.03369.x 20210806

[96] Moulton VR Suarez-Fueyo A Meidan E Li H Mizui M Tsokos GC. Pathogenesis of human systemic lupus erythematosus: a cellular perspective. Trends Mol Med. (2017) 23:615–35. 10.1016/j.molmed.2017.05.006 28623084 PMC5650102

[97] Hall JC Casciola-Rosen L Rosen A. Altered structure of autoantigens during apoptosis. Rheum Dis Clin North Am. (2004) 30:455–71 vii. 10.1016/j.rdc.2004.04.012 15261336

[98] Vollmer J Tluk S Schmitz C Hamm S Jurk M Forsbach A. Immune stimulation mediated by autoantigen binding sites within small nuclear RNAs involves Toll-like receptors 7 and 8. J Exp Med. (2005) 202:1575–85. 10.1084/jem.20051696 16330816 PMC2213330

[99] Means TK Latz E Hayashi F Murali MR Golenbock DT Luster AD. Human lupus autoantibody-DNA complexes activate DCs through cooperation of CD32 and TLR9. J Clin Invest. (2005) 115:407–17. 10.1172/JCI23025 15668740 PMC544604

[100] Vallin H Perers A Alm GV Ronnblom L. Anti-double-stranded DNA antibodies and immunostimulatory plasmid DNA in combination mimic the endogenous IFN-alpha inducer in systemic lupus erythematosus. J Immunol. (1999) 163:6306–13. 10570325

[101] Eloranta ML Lovgren T Finke D Mathsson L Ronnelid J Kastner B. Regulation of the interferon-alpha production induced by RNA-containing immune complexes in plasmacytoid dendritic cells. Arthritis Rheum. (2009) 60:2418–27. 10.1002/art.24686 19644885

[102] Baiu DC Barger B Sandor M Fabry Z Hart MN. Autoantibodies to vascular smooth muscle are pathogenic for vasculitis. Am J Pathol. (2005) 166:1851–60. 10.1016/S0002-9440(10)62494-7 15920169 PMC1602413

[103] Tsokos GC Lo MS Costa Reis P Sullivan KE. New insights into the immunopathogenesis of systemic lupus erythematosus. Nat Rev Rheumatol. (2016) 12:716–30. 10.1038/nrrheum.2016.186 27872476

[104] Kurts C Panzer U Anders HJ Rees AJ. The immune system and kidney disease: basic concepts and clinical implications. Nat Rev Immunol. (2013) 13:738–53. 10.1038/nri3523 24037418

[105] Nimmerjahn F Ravetch JV. Fcgamma receptors as regulators of immune responses. Nat Rev Immunol. (2008) 8:34–47. 10.1038/nri2206 18064051

[106] de Zubiria Salgado A Herrera-Diaz C Lupus nephritis: an overview of recent findings. Autoimmune Dis. (2012) 2012:849684 10.1155/2012/849684 22536486 PMC3318208

[107] Voet S Prinz M van Loo G. Microglia in central nervous system inflammation and multiple sclerosis pathology. Trends Mol Med. (2019) 25:112–23. 10.1016/j.molmed.2018.11.005 30578090

[108] Goverman J. Autoimmune T cell responses in the central nervous system. Nat Rev Immunol. (2009) 9:393–407. 10.1038/nri2550 19444307 PMC2813731

[109] Mars LT Saikali P Liblau RS Arbour N. Contribution of CD8 T lymphocytes to the immuno-pathogenesis of multiple sclerosis and its animal models. Biochim Biophys Acta. (2011) 1812:151–61. 10.1016/j.bbadis.2010.07.006 20637863 PMC5052066

[110] Steinman RM Banchereau J. Taking dendritic cells into medicine. Nature. (2007) 449:419–26. 10.1038/nature06175 17898760

[111] Calderon TM Eugenin EA Lopez L Kumar SS Hesselgesser J Raine CS. A role for CXCL12 (SDF-1alpha) in the pathogenesis of multiple sclerosis: regulation of CXCL12 expression in astrocytes by soluble myelin basic protein. J Neuroimmunol. (2006) 177:27–39. 10.1016/j.jneuroim.2006.05.003 16782208

[112] Krumbholz M Theil D Cepok S Hemmer B Kivisakk P Ransohoff RM. Chemokines in multiple sclerosis: CXCL12 and CXCL13 up-regulation is differentially linked to CNS immune cell recruitment. Brain. (2006) 129:200–11. 10.1093/brain/awh680 16280350

[113] Grip O Bredberg A Lindgren S Henriksson G. Increased subpopulations of CD16(+) and CD56(+) blood monocytes in patients with active Crohn's disease. Inflamm Bowel Dis. (2007) 13:566–72. 10.1002/ibd.20025 17260384

[114] Kaser A Zeissig S Blumberg RS. Inflammatory bowel disease. Annu Rev Immunol. (2010) 28:573–621. 10.1146/annurev-immunol-030409-101225 20192811 PMC4620040

[115] Brenchley JM Price DA Schacker TW Asher TE Silvestri G Rao S. Microbial translocation is a cause of systemic immune activation in chronic HIV infection. Nat Med. (2006) 12:1365–71. 10.1038/nm1511 17115046

[116] Moir S Chun TW Fauci AS. Pathogenic mechanisms of HIV disease. Annu Rev Pathol. (2011) 6:223–48. 10.1146/annurev-pathol-011110-130254 21034222

[117] Aukrust P Muller F Lien E Nordoy I Liabakk NB Kvale D. Tumor necrosis factor (TNF) system levels in human immunodeficiency virus-infected patients during highly active antiretroviral therapy: persistent TNF activation is associated with virologic and immunologic treatment failure. J Infect Dis. (1999) 179:74–82. 10.1086/314572 9841825

[118] Yeap WH Wong KL Shimasaki N Teo EC Quek JK Yong HX. CD16 is indispensable for antibody-dependent cellular cytotoxicity by human monocytes. Sci Rep. (2016) 6:34310. 10.1038/srep34310 27670158 PMC5037471

[119] Lamarthee B de Vassoigne F Malard F Stocker N Boussen I Mediavilla C. Quantitative and functional alterations of 6-sulfo LacNac dendritic cells in multiple myeloma. Oncoimmunology. (2018) 7:e1444411. 10.1080/2162402X.2018.1444411 29900053 PMC5993501

[120] Perez-Aso M Montesinos MC Mediero A Wilder T Schafer PH Cronstein B. Apremilast, a novel phosphodiesterase 4 (PDE4) inhibitor, regulates inflammation through multiple cAMP downstream effectors. Arthritis Res Ther. (2015) 17:249. 10.1186/s13075-015-0771-6 26370839 PMC4570588

[121] Schafer PH Parton A Gandhi AK Capone L Adams M Wu L. Apremilast, a cAMP phosphodiesterase-4 inhibitor, demonstrates anti-inflammatory activity *in vitro* and in a model of psoriasis. Br J Pharmacol. (2010) 159:842–55. 10.1111/j.1476-5381.2009.00559.x 20050849 PMC2829210

[122] Cutolo M Myerson GE Fleischmann RM Liote F Diaz-Gonzalez F Van den Bosch F. A phase III, randomized, controlled trial of apremilast in patients with psoriatic arthritis: results of the PALACE 2 trial. J Rheumatol. (2016) 43:1724–34. 10.3899/jrheum.151376 27422893

[123] Saporito RC Cohen DJ. Apremilast use for moderate-to-severe atopic dermatitis in pediatric patients. Case Rep Dermatol. (2016) 8:179–84. 10.1159/000446836 27504087 PMC4965536

[124] Oehrl S Olaru F Kunze A Maas M Pezer S Schmitz M. Controlling the pro-inflammatory function of 6-sulfo LacNAc (slan) dendritic cells with dimethylfumarate. J Dermatol Sci. (2017) 87:278–84. 10.1016/j.jdermsci.2017.06.016 28732748

[125] Maier C Ramming A Bergmann C Weinkam R Kittan N Schett G. Inhibition of phosphodiesterase 4 (PDE4) reduces dermal fibrosis by interfering with the release of interleukin-6 from M2 macrophages. Ann Rheumat Dis. (2017) 76:1133–41. 10.1136/annrheumdis-2016-210189 28209630

[126] Kocieda VP Adhikary S Emig F Yen JH Toscano MG Ganea D. Prostaglandin E2-induced IL-23p19 subunit is regulated by cAMP-responsive element-binding protein and C/AATT enhancer-binding protein beta in bone marrow-derived dendritic cells. J Biol Chem. (2012) 287:36922–35. 10.1074/jbc.M112.402958 22977257 PMC3481295

[127] Bros M Montermann E Cholaszczynska A Reske-Kunz AB. The phosphodiesterase 4 inhibitor roflumilast augments the Th17-promoting capability of dendritic cells by enhancing IL-23 production, and impairs their T cell stimulatory activity due to elevated IL-10. Int Immunopharmacol. (2016) 35:174–84. 10.1016/j.intimp.2016.03.025 27070502

[128] Al-Jaderi Z Maghazachi AA. Utilization of dimethyl fumarate and related molecules for treatment of multiple sclerosis, cancer, and other diseases. Front Immunol. (2016) 7:278. 10.3389/fimmu.2016.00278 27499754 PMC4956641

[129] Gavrilescu LC Denkers EY. Interleukin-12 p40- and Fas ligand-dependent apoptotic pathways involving STAT-1 phosphorylation are triggered during infection with a virulent strain of Toxoplasma gondii. Infect Immunity. (2003) 71:2577–83. 10.1128/IAI.71.5.2577-2583.2003 12704131 PMC153288

[130] Wehner R Bitterlich A Meyer N Kloss A Schakel K Bachmann M. Impact of chemotherapeutic agents on the immunostimulatory properties of human 6-sulfo LacNAc+ (slan) dendritic cells. Int J Cancer. (2013) 132:1351–9. 10.1002/ijc.27786 22907335

[131] Cavo M. Proteasome inhibitor bortezomib for the treatment of multiple myeloma. Leukemia. (2006) 20:1341–52. 10.1038/sj.leu.2404278 16810203

[132] Richardson PG Mitsiades C Hideshima T Anderson KC. Bortezomib: proteasome inhibition as an effective anticancer therapy. Annu Rev Med. (2006) 57:33–47. 10.1146/annurev.med.57.042905.122625 16409135

[133] Straube C Wehner R Wendisch M Bornhauser M Bachmann M Rieber EP. Bortezomib significantly impairs the immunostimulatory capacity of human myeloid blood dendritic cells. Leukemia. (2007) 21:1464–71. 10.1038/sj.leu.2404734 17495970

